# Supplementary material for: The effects of haloperidol on motor vigour and movement fusion during sequential reaching
Source: PLoS One. 2025 Jan 31;20(1):e0316894. doi: 10.1371/journal.pone.0316894 (PMC11785334; doi:10.1371/journal.pone.0316894)
Supplement: S1 Appendix — LMMs were conducted in JASP and included four fixed effects: 1. Reward (1 = Reward; 2 = No reward), 2. Drug (1 = haloperidol; 2 = placebo/control), 3. Working Memory (WM; 1 = high; 2 = low) and 4. Trial Number (1:180). In the analysis, low WM (for the WM variable), No Reward (for the Reward variable), and haloperidol (for the Drug variable) were coded as the reference categories (i.e., 0). Therefore, looking at S1 Table in S1 Appendix, the results show that the Reward groups (Rew) produced higher maximum velocity (velmax) compared to the No Reward (NoRew) groups. Additionally, the significant interaction between Trial Number and Drug indicates that the haloperidol groups (Halo) exhibited a decrease in velmax over the course of training compared to the Ctrl groups (since placebo/control is coded as 0). (DOCX) [file pone.0316894.s002.docx]

**Supplementary** **Table 1. Mixed-effect model for vel_max_ for *Day1***

Model: vel_peak_ ~ Trial * Drug * Reward * WM + (Trial Number | Participant)

AIC: 87099 BIC: 87253 Log-likelihood: -43529 Deviance: 87064

| **Variable** | **Estimate** | **SE** | **df** | **t** | **p** |
| --- | --- | --- | --- | --- | --- |
| Intercept | 27.88 | 0.639 | 84 | 43.607 | < .001 |
| WM | -0.862 | 0.639 | 84 | -1.348 | 0.181 |
| Trial Number | 1.113 | 0.351 | 84 | 3.166 | **0.002** |
| Reward | 4.063 | 0.639 | 84 | 6.356 | **< .001** |
| Drug | 0.597 | 0.639 | 84 | 0.934 | 0.353 |
| WM * Trial Number | 0.613 | 0.351 | 84 | 1.743 | 0.085 |
| WM * Reward | -0.605 | 0.639 | 84 | -0.947 | 0.346 |
| Trial Number * Reward | -0.163 | 0.351 | 84 | -0.463 | 0.645 |
| WM * Drug | -0.33 | 0.639 | 84 | -0.516 | 0.607 |
| Trial Number * Drug | -0.702 | 0.351 | 84 | -1.997 | **0.049** |
| Reward * Drug | 0.174 | 0.639 | 84 | 0.273 | 0.786 |
| WM * Trial Number * Reward | 0.197 | 0.351 | 84 | 0.559 | 0.577 |
| WM * Trial Number * Drug | -0.669 | 0.351 | 84 | -1.904 | 0.06 |
| WM * Reward * Drug | 0.749 | 0.639 | 84 | 1.171 | 0.245 |
| Trial Number * Reward * Drug | -0.71 | 0.351 | 84 | -2.02 | **0.047** |
| WM * Trial Number * Reward * Drug | -0.685 | 0.351 | 84 | -1.949 | 0.055 |

**Supplementary** **Table 2. Mixed-effect model for vel_max_ for *Day2***

Model: vel_peak_ ~ Trial Number * Drug * Reward * WM + (Trial Number | Participant)

AIC: 88154 BIC: 88154 Log-likelihood: -44057 Deviance: 88118

| **Variable** | **Estimate** | **SE** | **df** | **t** | **p** |
| --- | --- | --- | --- | --- | --- |
| Intercept | 31.808 | 0.706 | 84 | 45.081 | **< .001** |
| WM | -0.458 | 0.706 | 84 | -0.65 | 0.518 |
| Trial Number | 1.018 | 0.288 | 84 | 3.538 | **< .001** |
| Reward | 3.239 | 0.706 | 84 | 4.59 | **< .001** |
| Drug | -1.07 | 0.706 | 84 | -1.517 | 0.133 |
| WM * Trial Number | 0.204 | 0.288 | 84 | 0.708 | 0.481 |
| WM * Reward | -1.001 | 0.706 | 84 | -1.419 | 0.16 |
| Trial Number * Reward | 0.276 | 0.288 | 84 | 0.96 | 0.34 |
| WM * Drug | -1.252 | 0.706 | 84 | -1.775 | 0.08 |
| Trial Number * Drug | -0.004 | 0.288 | 84 | -0.015 | 0.988 |
| Reward * Drug | -0.756 | 0.706 | 84 | -1.071 | 0.287 |
| WM * Trial Number * Reward | 0.403 | 0.288 | 84 | 1.4 | 0.165 |
| WM * Trial Number * Drug | 0.17 | 0.288 | 84 | 0.59 | 0.557 |
| WM * Reward * Drug | -0.305 | 0.706 | 84 | -0.432 | 0.667 |
| Trial Number * Reward * Drug | 0.322 | 0.288 | 84 | 1.12 | 0.266 |
| WM * Trial Number * Reward * Drug | 0.248 | 0.288 | 84 | 0.863 | 0.39 |
|  |  |  |  |  |  |

**Supplementary** **Table 3. Mixed-effect model for FI for *Day1***

Model: FI ~ Trial Number * Drug * Reward * WM + (Trial Number | Participant)

AIC: 16602 BIC: 16757 Log-likelihood: -8281 Deviance: 16497

| **Variable** | **Estimate** | **SE** | **df** | **t** | **p** |
| --- | --- | --- | --- | --- | --- |
| Intercept | 1.073 | 0.072 | 84 | 14.908 | **<0 .001** |
| WM | 0.125 | 0.072 | 84 | 1.732 | 0.087 |
| Trial Number | 0.219 | 0.037 | 84 | 5.879 | **< 0.001** |
| Reward | 0.235 | 0.072 | 84 | 3.269 | **0.002** |
| Drug | 0.121 | 0.072 | 84 | 1.683 | 0.096 |
| WM * Trial Number | <0.001 | 0.037 | 84 | 0.021 | 0.983 |
| WM * Reward | 0.128 | 0.072 | 84 | 1.776 | 0.079 |
| Trial Number * Reward | 0.028 | 0.037 | 84 | 0.76 | 0.45 |
| WM * Drug | 0.007 | 0.072 | 84 | 0.094 | 0.926 |
| Trial Number * Drug | -0.056 | 0.037 | 84 | -1.503 | 0.137 |
| Reward * Drug | 0.056 | 0.072 | 84 | 0.783 | 0.436 |
| WM * Trial Number * Reward | 0.023 | 0.037 | 84 | 0.629 | 0.531 |
| WM * Trial Number * Drug | -0.075 | 0.037 | 84 | -2.002 | **0.049** |
| WM * Reward * Drug | 0.155 | 0.072 | 84 | 2.154 | **0.034** |
| Trial Number * Reward * Drug | 0.007 | 0.037 | 84 | 0.178 | 0.859 |
| WM * Trial Number * Reward * Drug | -0.048 | 0.037 | 84 | -1.281 | 0.204 |

**Supplementary** **Table 4. Mixed-effect model for FI for *Day2***

Model: FI ~ Trial Number * Drug * Reward * WM + (Trial Number | Participant)

AIC: 9068 BIC: 9223 Log-likelihood: -4514 Deviance: 8961

| **Variable** | **Estimate** | **SE** | **df** | **t** | **p** |
| --- | --- | --- | --- | --- | --- |
| Intercept | 1.615 | 0.095 | 84 | 16.979 | **< .001** |
| WM | 0.075 | 0.095 | 84 | 0.786 | 0.434 |
| Trial Number | 0.093 | 0.025 | 84 | 3.675 | **< .001** |
| Reward | 0.266 | 0.095 | 84 | 2.798 | **0.006** |
| Drug | 0.038 | 0.095 | 84 | 0.397 | 0.692 |
| WM * Trial Number | 0.027 | 0.025 | 84 | 1.071 | 0.287 |
| WM * Reward | 0.045 | 0.095 | 84 | 0.478 | 0.634 |
| Trial Number * Reward | 0.049 | 0.025 | 84 | 1.947 | 0.055 |
| WM * Drug | -0.088 | 0.095 | 84 | -0.928 | 0.356 |
| Trial Number * Drug | -0.004 | 0.025 | 84 | -0.14 | 0.889 |
| Reward * Drug | 0.081 | 0.095 | 84 | 0.849 | 0.399 |
| WM * Trial Number * Reward | 0.036 | 0.025 | 84 | 1.412 | 0.162 |
| WM * Trial Number * Drug | 0.028 | 0.025 | 84 | 1.11 | 0.27 |
| WM * Reward * Drug | 0.062 | 0.095 | 84 | 0.656 | 0.513 |
| Trial Number * Reward * Drug | 0.031 | 0.025 | 84 | 1.225 | 0.224 |
| WM * Trial Number * Reward * Drug | -0.001 | 0.025 | 84 | -0.055 | 0.956 |

**Supplementary** **Table 5. Mixed-effect model for MT for *Day1***

Model: MT ~ Trial Number * Drug * Reward * WM + (Trial Number | Participant)

AIC: 27675 BIC: 27829 Log-likelihood: -13817 Deviance: 27577

| **Variable** | **Estimate** | **SE** | **df** | **t** | **p** |
| --- | --- | --- | --- | --- | --- |
| Intercept | 4.465 | 0.099 | 84 | 45.007 | **< .001** |
| WM | 0.05 | 0.099 | 84 | 0.509 | 0.612 |
| Trial Number | -0.266 | 0.047 | 84 | -5.614 | **< .001** |
| Reward | -0.671 | 0.099 | 84 | -6.766 | **< .001** |
| Drug | -0.113 | 0.099 | 84 | -1.139 | 0.258 |
| WM * Trial Number | -0.057 | 0.047 | 84 | -1.199 | 0.234 |
| WM * Reward | 0.003 | 0.099 | 84 | 0.033 | 0.974 |
| Trial Number * Reward | 0.08 | 0.047 | 84 | 1.687 | 0.095 |
| WM * Drug | 0.064 | 0.099 | 84 | 0.643 | 0.522 |
| Trial Number * Drug | 0.097 | 0.047 | 84 | 2.054 | **0.043** |
| Reward * Drug | -0.025 | 0.099 | 84 | -0.256 | 0.799 |
| WM * Trial Number * Reward | -0.01 | 0.047 | 84 | -0.218 | 0.828 |
| WM * Trial Number * Drug | 0.062 | 0.047 | 84 | 1.316 | 0.192 |
| WM * Reward * Drug | -0.192 | 0.099 | 84 | -1.934 | 0.056 |
| Trial Number * Reward * Drug | 0.07 | 0.047 | 84 | 1.485 | 0.141 |
| WM * Trial Number * Reward * Drug | 0.064 | 0.047 | 84 | 1.344 | 0.183 |

**Supplementary** **Table 6. Mixed-effect model for MT for *Day2***

Model: MT ~ Trial Number * Drug * Reward * WM + (Trial Number | Participant)

AIC: 19057 BIC: 19212 Log-likelihood: -9508 Deviance: 18951

| **Variable** | **Estimate** | **SE** | **df** | **t** | **p** |
| --- | --- | --- | --- | --- | --- |
| Intercept | 3.631 | 0.086 | 84 | 42.232 | **< .001** |
| WM | 0.025 | 0.086 | 84 | 0.295 | 0.769 |
| Trial Number | -0.142 | 0.032 | 84 | -4.485 | **< .001** |
| Reward | -0.442 | 0.086 | 84 | -5.136 | **< .001** |
| Drug | 0.086 | 0.086 | 84 | 0.995 | 0.322 |
| WM * Trial Number | -0.043 | 0.032 | 84 | -1.343 | 0.183 |
| WM * Reward | 0.06 | 0.086 | 84 | 0.7 | 0.486 |
| Trial Number * Reward | 0.008 | 0.032 | 84 | 0.241 | 0.81 |
| WM * Drug | 0.124 | 0.086 | 84 | 1.448 | 0.151 |
| Trial Number * Drug | 0.003 | 0.032 | 84 | 0.088 | 0.93 |
| Reward * Drug | 0.007 | 0.086 | 84 | 0.08 | 0.937 |
| WM * Trial Number * Reward | -0.059 | 0.032 | 84 | -1.849 | 0.068 |
| WM * Trial Number * Drug | -0.04 | 0.032 | 84 | -1.249 | 0.215 |
| WM * Reward * Drug | -0.047 | 0.086 | 84 | -0.551 | 0.583 |
| Trial Number * Reward * Drug | -0.026 | 0.032 | 84 | -0.832 | 0.408 |
| WM * Trial Number * Reward * Drug | -0.029 | 0.032 | 84 | -0.917 | 0.362 |
