# Supplementary material for: The effects of haloperidol on motor vigour and movement fusion during sequential reaching
Source: PLoS One. 2025 Jan 31;20(1):e0316894. doi: 10.1371/journal.pone.0316894 (PMC11785334; doi:10.1371/journal.pone.0316894)
Supplement: Attachment — Submitted filename: Response to Reviewers.docx [file pone.0316894.s003.docx]

Summary of key points for editors

We added three sections to the discussion to address limitations, discuss relevant literature in more detail and map out new research avenues

We have rephrased our wording in the result section

Colour code

Referee comments in blue

Answers in red

*New wording in green italics*

Referee 1:

1) Consider chaning haloperidol to "a dopamine antagonist" in the title

We thank the reviewer for this suggestion have changed the title of the manuscript.

*The effects of haloperidol on motor vigour and movement fusion during sequential reaching*

2) Line 33 - abstract -consider mentioning that this task has previously be used to show reward-induced differences in vigour and fusion

We agree with the reviewer and have changed the first sentence to make this clearer.

*Reward is a powerful tool to enhance human motor behaviour with previous research showing that during a sequential reaching movement, a monetary incentive leads to increased speed of each movement (motor vigour effect), whilst reward-based performance feedback increases the speed of transition between movements (movement fusion effect).*

3) line 36-37 and line 91 - reward and no reward → probably reward or no reward would be clearer. I think it would make more sense to say there are four groups (2*2 design)

We agree with the reviewer and have changed the sentence to make this clearer.

*95 participants were randomly assigned to a reward or no reward group and were given either 2.5mg of the dopamine antagonist haloperidol or a placebo.*

4) line 71 - explain what a D2 antagonist is

We agree with the reviewer and have added the following paragraph.

*Conversely, research using haloperidol demonstrated a reduction in motor vigour during reward-effort decision-making (26). Haloperidol is a D2-receptor antagonist that shows a limited affinity to D1 receptors and has superior in vivo D2 binding. It blocks DA D2 binding in the basal ganglia (BG) but not in the prefrontal cortex and as such can be considered to selectively modulate DA levels within the BG pathway (44).*

5) line 94 - better to write the number of males and females (rather than assuming all non-males are females)

We agree with the reviewer and have added the number of self-identified females.

*[…] (42 males and 53 females; age range 18 - 42) […]*

6) line 96 - the two sentences starting with "All participants were ..." are repetitive, only one is needed (better the 2nd one)

We agree with the reviewer and have changed the second sentence to the following:

*Participants were pre-screened and were […]*

7) line 103 - did any of the participants not receive medical approval? it is not obvious what the MD was checking

Yes, some participants did not receive medical approval and were excluded from the study.

8) line 104 - 7 were left-handed, were all the rest right-handed (or were some ambidexterous?)

We agree with the reviewer and included more information in the text.

*[…] self-reportedly right-handed (N = 88) while the rest was left-handed (N = 7) and gave […]*

9) line 107 - maybe you mean counterbalanced rather than pseudorandomly (to ensure similar group sizes?)

Yes, the study was counterbalanced! Thank you

*Before the start of the experiment, participants were counterbalanced to one of the available groups.*

10) line 174 - "and hence the box remained block" → it is not obvious which one results from the other

Yes, it should say:

*In contrast, participants that were in a NoRew groups were told to move as fast and accurately as possible and here the start box remained black.*

11) line 193 - what instructions were given during the "learning" phase (maybe familiarization is a better term?)

We agree with the reviewer and changed that to ‘Familiarisation’.

12) Figure 1 e - in my experience it is rare for "complete" fusion to take place (at least in a single day), maybe this would be clearer if an overlapping velocity profile was shown

We agree with the reviewer that ‘complete fusion’ is rare to take place over the course of one day. But here we just wanted to illustrate what ‘complete fusion’ would look like.

13) line 239 - VWM - define what this is

We thank the reviewer for this and have defined that in the text.

*i) Illustration of Verbal Working Memory (VWM) task.*

14) line 269 - did you indeed find that there was a separation into "low" and "high" WM, i.e. did the distribution have two peaks rather than a single peak? Why should be expect low and high to both consist of exactly half the participants?

We thank the reviewer and added to the text that the separation into ‘low’ and ‘high’ was based on a median split similar to Frank MJ, O’Reilly RC. (2006).

*WM Perf was used to identify participants with low and high WM (based on a median split) to be included in the subsequent analysis*

15) line 285 - there seems to be some contradiction here - MT is defined as the time from exiting the start box, whereas reaction time is when the start position exceeds 2cm (also it is not clear - exceeds 2cm from what?)

We thank the reviewer for spotting this! It should say ‘includes’!

*This includes reaction time, which describes the time between target appearance and when the participants’ start position exceeded 2cm.*

16) line 290 - 291 - it is not clear how the segments are divided - within 2cm define the start or end? first or last time?

Yes, we agree that this is not clear and amended the manuscript to make this more clear.

*The velocity profile was then divided into segments representing movements to each individual target (8 segments). Specifically, a segment was defined as when the cursor left the target until it left the next. Note here that the final segment was defined as when the cursor left the previous target until it reached the final target.*

17) line 297 - 298 - the definition of fusion (stop period between 2 movements gradually disappearing) is not clear - at some point they no longer stop but the velocity trough slowly increases over time (rather than the stop period gradually disappearing)

Yes, we agree that this is not clear and amended the manuscript to make this more clear.

*This is represented in the velocity profile by the velocity trough between the two movements gradually increasing*

18) line 307 - the explanation in the text of the fusion index differs somewhat from the equation (equation 2), which corresponds to a single "trough" whereas the text refers to 7 troughs. To make them equivalent, the equation could be changed to include a sum of i from 1 to 7, and then change 1 to i, and 2 to i+1 (and something similar for v_min)

Yes, we agree that this is not clear and amended the manuscript to make this more clear.

*Given that in this task seven transitions had to be completed, we calculated the fusion index separately for each transition, which led to the maximum FI value being 7 in each trial.*

19) line 308 - data exclusion - did you consider removing the trials also after "problematic" trials? These are likely also affected by the "problematic" trial or the dual task

This is an interesting suggestion! We have not considered removing the following trials but considering that only 11 trials were considered as problematic, we believe that this approach would not change the results significantly.

20) line 382 - do you mean your measures were non-normal (rather than nonparametric), and hence you used nonparametric methods?
Yes, absolutely! Thanks for spotting this!

*We used one-sample Kolmogorov–Smirnov tests to test our data for normality and found that all measures were not normally distributed.*

21) line 400 - is this analysis (until the end of the paragraph) now on the high WM group? it is not clear
Yes, we agree that this is not clear and amended the manuscript to make this more clear.

*Specifically, in line with previous work, we conducted separate analysis for low and high WM participants using a median split and only included the low WM participants in the analysis (44).*

22) line 414 - "we found no differences" - p>0.05 does not mean there are no differences, to make this claim a different test is needed (e.g. Bayesian ANOVA or two one-sided t-tests)
Yes, we agree with the reviewer and changed the wording.

*We found no significant differences at Baseline*

23) line 453 - the line ended without closing the brackets
Thank you for spotting this. We changed it.

24) line 488 - in the first paragraph of the discussion it may be helpful to remind the reader what FI is

Yes, we agree with the reviewer and changed the wording.
*In contrast, performance-based feedback enhanced movement fusion indexed as increases in fusion (FI).*

25) line 508 (or elsewhere in the discussion) - it should be considered here or elsewhere why max vel and MT are not mirror images of each other. They are fairly close to mirror (see Figure 2 c and g), but where they differ may lead to some insight on what is happening here. For example, rather than increasing overlap between "submovements", there may instead be a "new" motor primitive created (as in Sosnik et al., 2004), which may mean the the FI is not the ideal measure
*This is an interesting suggestion. Within this context, we previously used a submovement analysis and compared it to the fusion index with results indicating a perfect match between the two.*

26) line 523 - are fusion and chunking the same thing necessarily?

No, we do not believe they are necessarily the same thing. However, chunking and fusion and equally co-articulation all represent optimisation processes that allow for a more efficient transition between sequence elements. Historically, co-articulation has been associated with speech production, while chunking has often been used within the context of more discrete sequences. Finally, we have introduced fusion within the context of more continuous arm movements. Therefore, they do potentially refer to different end effectors but describe similar processes. We did not want to suggest that they are the same thing and have changed the manuscript to reflect that more accurately.
*DA has been implicated to underlie movement fusion and chunking with evidence coming*

27) line 527 - remind the reader what tiapride does

Yes, we agree with the reviewer and added more information.
*Post-hoc analyses aimed to further investigate the significant interaction between Drug*Reward*WM did not produce any significant results, which diverges from previous work that found impaired motor sequence learning during a button-press task using the D2-antagonist tiapride (36).*

28) line 548 - the paper would be improved by introduction of a limitations section (particularly given the many surprising null results).

We have revised both the results section and the discussion/abstract in light of new findings derived from an analysis, including marginal effects from the LMM as suggested by Referee2. Our updated results now demonstrate that haloperidol does impair the reward-based effects on motor vigour, aligning with previous research. However, we did not find strong evidence that haloperidol influences movement fusion, which contrasts with prior findings. We have addressed possible reasons for this discrepancy in the discussion. Please refer to our response to Referee 2, point 1, for further details on the changes we made.

Referee 2:

1) Throughout the manuscript and in the Discussion, the authors refer to the subtle/small effect of Haloperidol on the modulation of motor vigour/peak velocity. But no effect sizes are estimated. From the significant LMM interaction effect Drug * Trial *Reward, it would be good to assess marginal effects of Drug and Reward on the slope (DV by trial). I understand the statement “small effect” comes from doing trial-wise permutation tests and observing the effects exclusively in trials 170-180, but assessing the LMM slopes in the marginal effects of the three way-interaction seems more suitable.

We thank the reviewer for pointing out that the marginal effects can shed light on significant three-way interaction! We have looked at the marginal effects and they strengthen our conclusions that haloperidol did cancel out the reward-based effects on vel_max_. We have re-written the manuscript accordingly:

*Abstract:
We did not find evidence that haloperidol affected the facilitatory effects of reward on movement fusion. However, we found that haloperidol negated the reward-based effects on motor vigour.*

*Therefore, our results suggest that a D2-antagonist differentially influences reward-based effects on movement vigour and movement fusion, indicating that the dopaminergic mechanisms underlying these two processes may be distinct.*

*Methods:*
*Significant interactions that included Trial Number were further analysed by calculating the marginal effects of the fixed effects on the slope of the dependent variable (across trials). The beta coefficients (β) of the slopes and their corresponding 95% confidence intervals (CI) were used to perform pairwise z-tests to assess whether slopes were significantly different. Multiple comparisons were corrected using Bonferroni correction. In contrast, significant interactions that did not include Trial Number were further assessed using a between-groups ANOVA using the median across all trials for each participant as input. Furthermore, to determine during which trial window groups were significantly different, we used a row-shuffle non-parametric permutation test.*

*Results:*
*Estimating marginal effects, we found that Ctrl-R significantly increased velmax over the course of Training (β = 2.4, CI [1.0 3.7], p < 0.001). In contrast, velmax decreased for Halo-R albeit not significantly (β = -0.46, CI [-1.76 0.85], p = 0.49). The slopes for NoRew groups were positive but not significant (Halo-NR: β = 1.28, CI [-0.15 2.7], p = 0.079; Ctrl-NR: β = 1.27, CI [-0.14 2.7], p = 0.077). Running pairwise z-test comparisons we found the slopes to be significantly different between Ctrl-R and Halo-R (p = 0.023) but did not find any other comparison to be significant. This suggests that haloperidol led to a selective decrease in reward-based motor vigour.*

*Crucially, we did not observe any performance differences between the NoRew groups (Figure 2e), which indicates that haloperidol selective~~, yet small,~~ effect on the reward-based effects on motor vigour.*

*Estimating marginal effects, we found that the Ctrl groups significantly increased velmax over the course of Training (β = 1.8, CI [0.8 2.8], p < 0.001), while the Halo groups did not (β = 0.41, CI [-0.6 1.4], p = 0.41). Results from the pairwise z-test comparison showed that the slopes were significantly different (z = -1.99; p = 0.047). This suggests that haloperidol led to a global decrease in motor vigour, which was most pronounced in the Halo-R group.*

*Estimating marginal effects, we found that the participants with high WM that received haloperidol did not improve in FI over the course of Training (β = 0.09, CI [-0.1 0.2], p = 0.240). In contrast all other groups did (Halo low WM: β = 0.24, CI [0.1 0.4], p = 0.001; Ctrl high WM: β = 0.35, CI [0.2 0.5], p < 0.001; Ctrl low WM: β = 0.20, CI [0.04 0.35], p = 0.012). Post-hoc analysis including pairwise z-test comparisons revealed no significant differences between slopes. The results suggest that haloperidol does not affect the faciliatory effect of reward on movement fusion. Yet, it may have a subtle effect on movement fusion in participants with high WM.*

*Estimating marginal effects, we found that the Ctrl groups exhibited a significant decrease in MTs over the course of Training (β = -0.37, CI [-0.5 -0.2], p < 0.001). The Halo group also showed a decrease in MTs, but to a lesser extent (β = 0.17, CI [-0.3 -0.04], p = 0.012). Post-hoc analysis revealed that the slopes were significantly different (z = 2.04, p = 0.041) suggesting that haloperidol led to a global slowing in the Halo groups. This aligns with the global slowing seen in velmax. Further […]*

*Discussion:*

*Furthermore, we found that haloperidol led to a selective impairment in motor vigour, which led to a global slowing in movement times. This result aligns with previous work showing that a D2 antagonist impairs motor vigour during reward-effort decision-making (26,44). Therefore, our results suggest that a D2-antagonist differentially influences reward-based effects on movement vigour and movement fusion, indicating that the dopaminergic mechanisms underlying these two processes may be distinct.*

*Our results showed that the D2 antagonist haloperidol led to a selective impairment on motor vigour. Specifically, we found that velmax decreased over the course of Training on Day1, whereas velmax significantly increased in Ctrl-R which is in line with previous work on the reward-based effects on motor vigour(10). Crucially, we did not observe any performance differences between the NoRew group. Thus, our results provide evidence that a D2 blockage impairs motor vigour which aligns with previous research(26). Specifically, previous work investigating the role of DA during reward-effort integration showed that a D2 antagonist reduces the willingness to produce effortful squeezes in a isometric grip force task(24,26,28). This is further in line with previous research showing that motor vigour can be enhanced with L-DOPA operationalising motor vigour as response speed(23). Therefore, previous and our results suggest that motor vigour relies on a dopaminergic mechanism that can be modulated with a D2 manipulation.*

*However, our results suggest that a D2-antagonist does not impair movement fusion. Specifically, we found that only participants with high WM that received haloperidol did not improve significantly in FI over the course of Training on Day1. However, post-hoc analysis showed that the rate of improvement in this group was not significantly different from the other groups. Additionally, our results do not show that haloperidol affects the reward-based effects on movement fusion. Post-hoc analyses aimed to further investigate the significant interaction between Drug*Reward*WM which only revealed a main effect for Reward. Therefore, our results did not find that haloperidol had a global effect on movement fusion, nor that it impairs the facilitatory effects of reward. This diverges from previous work that found impaired motor sequence learning during a button-press task using the D2-antagonist tiapride*

*In summary, in contrast to previous human work, we did not find strong evidence that a D2-antagonist modulates the reward-based effects on movement fusion during the production of sequential reaching movements. However, we found corroborating evidence that a D2-antagonist negates the reward-based effects on motor vigour. Taken together, our results suggest that a D2-antagonist differentially influences reward-based effects on movement vigour and movement fusion, indicating that the dopaminergic mechanisms underlying these two processes may be distinct.*

2) Response Accuracy analysis: Why not conduct a factorial analysis of RA including WM group, drug condition, and trial (Go/No-Go)?

We thank the reviewer for this comment. While we acknowledge that a factorial analysis could have been conducted, we opted to adhere closely to the original analysis pipeline established by Frank and O’Reilly (2006). Our primary goal was to establish a positive control to determine whether haloperidol was active during the experiment; therefore, we aimed to replicate their design as closely as possible.

3) Do the authors expect that reaction time is not modulated by haloperidol?

We have looked at reaction times before and we found that they are neither modulated by reward, haloperidol nor WM (see LMM results and corresponding figure below). We, therefore decided to only look at MT excluding reaction times from the analysis.

4) Please state the formula used in your analysis earlier, before specifying the implicit individual factor interactions implied by th e main LMM.

We now state the formula at the very beginning of the section:

*To investigate if haloperidol modulated the reward-based effects on vel_max_, we used linear mixed-models (LMM), which were performed separately for Training on Day1 (Training1) and Day2 (Training2) using the following formula:*

*Dependent Variable ~ 1 + Reward*Drug*WM*Trial Number + (Trial|Participant)*

5) When discussing the introduction of random effects, the text could be clearer. State that, given the design of your study, you expect that each subject contributes variability in the slope of changes in DV across trials. Here, changes across trials would reflect training effects (improvements in performance). Why not consider random effects of subjects on the intercept? Which model explains the data better? One including/excluding random effects of subjects on the slope of DV by trial?

We thank the reviewer for this comment and rephrased the text:

*In contrast, Trial Number was included as a random slope by participant because we expect that participants may show variable changes across trials; essentially reflecting differential improvements over the course of Training. Including Trial Number improved the model as seen in a lower AIC and BIC. Removing Trial as a random slope by participant increased the BIC and we therefore used this model for all LMMs presented in the paper. Similarly, including*

6) Please elaborate on this: “within LMM has been a heated topic (49).” Why is it a heated topic? Don’t LMMs already provide statistical effects on fixed effects, including main effects and interactions?

We have now removed this sentence because we have now included the marginal effects to further assess significant interactions. Again, thank you for suggesting this!

7) Why is this necessary? “This allowed us to investigate during which trials groups were exhibiting significantly different performance.” If participants performed 180 trials, do the authors expect to find between-group differences in some trials but not in others? Shouldn’t the collective behaviour of the DV across trials be simply parameterised as the intercept and slope? Comparing slopes between groups seems more meaningful than the DV on individual trials across 1:180.

We thank the reviewer for this comment and again for suggesting to look at the marginal effects of the fixed effects on the slope of the DV. This additional analysis confirmed that haloperidol indeed negated the reward-based effects on motor vigour. Moreover, the permutation analysis allowed us to show that, when comparing trials, this effect became significant by the end of Training on Day 1. This provides further detail regarding the time course of how haloperidol influenced the reward-related modulation of motor vigour.

*Significant interactions that included Trial Number were further analysed by calculating the marginal effects of fixed effects on the slope (across trials). Furthermore, to determine during which trial window groups were significantly different, we used a row-shuffle non-parametric permutation test.*

8) The post-hoc analyses following significant interaction effects should ideally be more extensive to assess all factor combinations and help us understand the implications of the interaction effect. For instance, page 16, lines 446-449: Would the interaction effect be explained by contrasting high and low WM groups separately for Reward groups (separately for each Drug group)?

We thank the reviewer for this suggestion! We ran a ANOVA with Reward, WM and Drug as between factors revealed a significant main effect for Reward (F = 6.42, p = 0.0022) but no other main effects or interactions were found. We added the following to the manuscript:
*An ANOVA* *with Reward, WM and Drug as between factors revealed a significant main effect for Reward (F = 6.42, p = 0.0022) but no other main effects or interactions were found.*

9) As mentioned, it is not clear why the permutation test separately for each trial is conducted, as effects of DV over time could be parametrised by the DV by trial slope, contingent on the other factors. The LMM could reveal these changes over time. Assessment of marginal effects could help here.

Thank you again for this suggestion. We have changed the analysis pipeline and amended the text (see above).

10) It would help interpret the LMM results if the estimates from the Supp tables were referenced to the coding of groups. For instance, which Drug / Reward groups are coded as 0, and which ones a 1? With that information, the authors could easily interpret e.g. the negative and significant Trial*Drug interaction for peak velocity (-0.702, p = 0.049). If Placebo is 0, then this interaction estimate indicates that Halo relative to Placebo, decreases the slope of the DV change over trials.

We thank the reviewer for this very constructive suggestion. This does help interpreting the supplementary tables. We added the following to the supplement:

*LMMs were conducted in JASP and included four fixed effects: 1. Reward (1 = Reward; 2 = No Reward), 2. Drug (1 = haloperidol; 2 = placebo), 3. Working Memory (WM; 1 = high; 2 = low) and 4. Trial Number (1:180). In the analysis, low WM (for the WM variable), No Reward (for the Reward variable), and haloperidol (for the Drug variable) were coded as the reference categories (i.e., 0). Therefore, looking at Table 1, the results show that the Reward groups (Rew) produced higher maximum velocity (velmax) compared to the No Reward (NoRew) groups. Additionally, the significant interaction between Trial and Drug indicates that the haloperidol groups (Halo) exhibited a decrease in velmax over the course of training compared to the Ctrl groups (since placebo/control is coded as 0).*

11) Discussion: Which evidence do the authors offer for this statement? “Nevertheless, our results highlight again that movement fusion is highly reward-sensitive sensitive, which suggests that there may be another dopaminergic mechanism underlining it that does not heavily rely on D2 DA availability.”

Based on the new findings, we removed this sentence from the manuscript. Yet, the only significant main effect identified in the post-hoc analysis was that of Reward, which is consistent with previous research showing that movement fusion is highly sensitive to reward (Sporn et al., 2022).

12) The summary statement should be revised as small effects of haloperidol on peak velocity/motor vigour and no effects on movement fusion is not the same as “we did not find strong evidence that a D2-antagonist modulates motor vigour and/or movement fusion during the production of sequential reaching movements.”

Based on the new findings, we changed the summary throughout the manuscript, which we believe aligns more with the findings inspired by your suggestions.

*In summary, in contrast to previous human work, we did not find strong evidence that a D2-antagonist modulates the reward-based effects on movement fusion during the production of sequential reaching movements. However, we found corroborating evidence that a D2-antagonist negates the reward-based effects on motor vigour. Taken together, our results suggest that a D2-antagonist differentially influences reward-based effects on movement vigour and movement fusion, indicating that the dopaminergic mechanisms underlying these two processes may be distinct.*

13) Figure 1 seems to have broken typography.

We thank the reviewer for spotting this. We have amended the figure.

*See Figure 1*

14) Figure 1e is unclear; the caption could be clearer. It seems that maximum velocity peaks are represented in the top left panel (blue traces), not in the top right panel. I do not see an illustration of '2) Fuse consecutive movements leading to increases in minimum velocities'. Which of the three panels shows an increase in minimum velocity? Why does the top right panel show only one movement instead of two, as shown in the background black traces?

We thank the reviewer for highlighting this issue. We have revised the figure caption and clarified the labelling of the strategies. The "Top Left" now refers to changes in peak velocities, while the "Top Right" refers to changes in movement fusion. We have also added arrows to indicate which parts of the velocity profile are affected: increases in peak velocities represent enhanced motor vigour, and increases in minimum velocities reflect improved movement fusion. In a perfectly fused movement, there is essentially only one movement peak, as the speed does not decrease when passing through the via point.

*See Figure 1*

15) Page 11, something is missing here: 'Importantly, similarly mixed-model ANOVA with Drug (haloperidol vs placebo) as between factors and Feedback Condition (positive vs negative) as a within factor and RA as the dependent variable.' It appears incomplete.

We thank the reviewer for highlighting this issue. We amended the text:

*To assess if the D2 antagonist haloperidol was active during the main experiment we used a mixed-model ANOVA with Drug (haloperidol vs placebo) as between factors and Feedback Condition (positive vs negative) as a within factor and RA as the dependent variable. Wilcoxon tests were employed when a significant interaction was reported.*

16) Page 13, distributions that do not meet the criteria for parametric statistical testing should indeed be analysed with non-parametric tests, but this sentence is not precise: 'We used one-sample Kolmogorov–Smirnov tests to test our data for normality and found that all measures were non-parametric.' Do the authors mean that 'and found that the data distributions were not suitable for parametric statistical testing, therefore …'?
We thank the reviewer for highlighting this issue. We amended the text:

*We used one-sample Kolmogorov–Smirnov tests to test our data for normality and found that all measures were not normally distributed. Median values were therefore used as input in all mixed-model ANOVAs (similar to(10,49)).*

17) Clarify what you mean by 'Trial Number' and 'Trial'. Are these separate variables?

Thanks for spotting this. We have skimmed through the manuscript and changed any *Trial* to Trial Number.

18) Line 397: better phrased as 'while it impaired…'?

We changed the text in light of the new results which led to this sentence being removed from the text.

19) Page 14, line 419: Where are the p-values from the permutation tests? Why do the authors argue that the effect was small/subtle? Did they estimate effect sizes?.

We now have included the marginal effects analysis which we think already addresses this point. Again, thanks for all your constructive feedback! This has really improved the manuscript.
